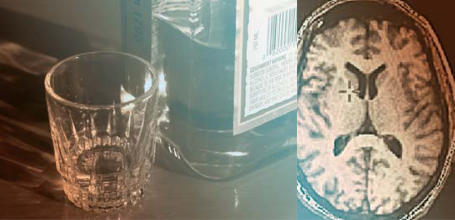# Environmental Connections: A Deeper Look into Mental Illness

**DOI:** 10.1289/ehp.115-a404

**Published:** 2007-08

**Authors:** Charles W. Schmidt

Mental illnesses produce some of the most challenging health problems faced by society, accounting for vast numbers of hospitalizations, disabilities resulting in billions in lost productivity, and sharply elevated risks for suicide. Scientists have long known that these potentially devastating conditions arise from combinations of genes and environmental factors. Genetic research has produced intriguing biological insights into mental illness, showing that particular gene variations predispose some individuals to conditions such as depression and schizophrenia.

Now, thanks to a growing union of epidemiology and molecular biology, the role of the environment in the etiology of mental illness has become more clear. Indeed, E. Fuller Torrey, president of the Treatment Advocacy Center, a nonprofit organization that promotes treatment advances in psychiatry, suggests that mental illnesses increasingly fall into the realm of environmental health. And from that platform, he says, new treatment advances could soon emerge.

“Some of the greatest advancements in twentieth-century medicine were achieved by identifying and preventing infectious diseases through vaccination, improved sanitary measures, improved nutrition, and diminished hazards of environmental contaminants,” adds Alan Brown, an associate professor of clinical psychiatry and epidemiology at Columbia University Medical Center. “If environmental risk factors for [mental illness] can be validated and confirmed, there is every reason to expect they will point to preventive measures that lower their risks and morbidity.”

## Everything But the Gene

Scientists define “environment” in the realm of mental illness broadly, some going so far as to suggest it encompasses everything that isn’t an inherited gene. That’s a departure from traditional thinking in environmental health, however, which has historically viewed environmental threats in the context of infectious agents, pollutants, and other exogenous factors that influence the individual’s physical surroundings. Environmental threats to mental health include these traditional parameters—along with pharmaceutical and illicit drugs, injuries, and nutritional deficiencies—but also consist of psychosocial conditions that relate to the individual’s perceptions of the social and physical world.

Any number of circumstances—for instance, sexual abuse, falling victim to crime, or the breakup of a relationship—can produce psychosocial stress. But experts assume each of these circumstances triggers more primal reactions, such as feelings of loss or danger, which serve to push victims toward a particular mental state. “Feelings of pure loss might lead to depressive disorders, while feelings of pure danger might lead to anxiety disorders,” explains Ronald Kessler, a professor of health care policy at Harvard Medical School. “And feelings of loss *and* danger might lead to both simultaneously.” Either alone or in combination, psychosocial and physiological stressors can interact with genetic vulnerability to alter brain chemistry and thus alter the individual’s mental health.

Several lines of evidence point to an environmental role in psychiatric disease. Among identical twins, if one becomes schizophrenic, the risk to the other is on average less than 50%, suggesting that environmental influences must somehow be involved. Similar findings have been observed with depression and other mental disorders.

Scientists have traditionally been challenged in their efforts to link mental illness with underlying causes, in part because the diseases are so amorphous, says Ezra Susser, a psychiatrist and department chair in epidemiology at the Columbia University Mailman School of Public Health. Unlike cancer or heart disease, which have clearly visible end points, mental disorders yield vague behaviors that vary widely among individuals. “They’re defined mainly by thoughts, behaviors, and feelings,” Susser says. “We don’t have biological measures on which to rest our diagnoses.”

Researchers and clinicians base psychiatric diagnoses on behavioral symptoms described in the *Diagnostic and Statistical Manual of Mental Disorders, Fourth Edition*, a handbook published by the American Psychiatric Association in 1994. However, many of the handbook’s 297 listed conditions share similar features, and patients typically show up with co-morbidities that obscure links to underlying risk factors. Schizophrenia, for example, is frequently accompanied by depression. Without being able to link exposures and outcomes more clearly, scientists have heretofore been unable to determine how environmental factors trigger psychiatric conditions, or why.

“If *environmental risk factors for [mental illness] can be validated and confirmed, there is every reason to expect they will point to preventive measures that lower their risks and morbidity.”*

–Alan Brown, Columbia University Medical Center

## A Glimpse of Biology

But now that’s starting to change. In a seminal study published 2 August 2002 in *Science*, Avshalom Caspi and Terrie Moffitt, who hold joint appointments as psychology professors at King’s College London and the University of Wisconsin–Madison, presented the first evidence linking genotype to mental illness through an environmental pathway. In so doing, they laid the foundations for more advanced studies of the environment’s role in mental illness. Specifically, Caspi and Moffitt found that maltreatment could induce antisocial personality disorder in children with a variant *MAOA* gene, which codes an enzyme that metabolizes neurotransmitters in the brain. That finding not only showed that genotype could influence an environmental risk factor’s capacity to induce mental illness, but also suggested that by curbing maltreatment, it might be possible to intervene in biological pathways that predispose some children to violence and crime.

Scientists *define “environment” in the realm of mental illness broadly, some going so far as to suggest it encompasses everything that isn’t an inherited gene.*

The next year, an article in the 18 July 2003 issue of *Science* showed that young people who go through emotionally stressful situations, such as losing a job or a romantic partner, are more prone to major depression if they inherit a variant form of the serotonin transporter gene, which participates in brain cell communication. Ironically, millions of people worldwide were already being treated for depression, anxiety, and other mood disorders with drugs that act on serotonin metabolism in a way that scientists still did not fully understand. Caspi and Moffitt’s discovery offered clues to that process that are still being investigated today. What they didn’t show, however, was how brain function might change in response to the gene variant.

That piece of the puzzle came from Daniel Weinberger, a branch chief at the National Institute of Mental Health, who used functional magnetic resonance imaging to demonstrate that individuals with the gene variant also demonstrated hyper-activity in the amygdala, a part of the brain that processes fear. Weinberger hypothesized that people with the variant are more likely to view the world as menacing. Therefore, he reasoned, the routine stresses of daily life could be amplified to the point of inducing depression.

Douglas Levinson, a professor of psychiatry at the Stanford University School of Medicine, describes the evolving literature on the variant serotonin transporter gene and its role in depression as “the first really interesting story to emerge from gene–environment research on mental illness.” But he adds that it’s still too early to know if the findings will hold up to further scrutiny. On the other hand, he says, “It could be that these interactions are more complex than our current knowledge allows us to imagine.”

Indeed, many other genes beyond the serotonin transporter have also been implicated in depression. For instance, in the February 2007 issue of the *American Journal of Psychiatry*, Levinson reported on a cluster of genes located on chromosome 15q that he suspects may link to depression by pathways that have nothing to do with serotonin. In the final analysis, he says, external agents probably interact with a variety of genes, each contributing a fraction to the overall risk. Moreover, some environmental exposures might be strong enough to trigger mental illness regardless of the individual’s genetic makeup.

Victor Carrion, an associate professor of psychiatry at Stanford University School of Medicine, suspects that is probably true of post-traumatic stress disorder (PTSD), a debilitating illness that follows terrifying experiences. “With most types of trauma, we see that thirty to fifty percent of individuals develop PTSD . . . and that suggests that genetic vulnerability plays a role,” he acknowledges. “But as the trauma become more severe—for instance, from kidnapping, torture, or sexual abuse—the prevalence rate can rise to nearly a hundred percent. So, that indicates environmental factors can double the prevalence, depending on severity [of the trauma].”

Carrion suggests in the March 2007 issue of *Pediatrics* that PTSD might be linked to excessive brain concentrations of cortisol, a steroid hormone. Released naturally during stress, cortisol at levels such as those produced during high stress kills neurons, including those in the hippocampus, a structure in the brain that participates in memory and emotion. Among children who have PTSD, the hippocampus is reduced in size, possibly because of cortisol-induced cell death, Carrion proposes. And that, he adds, offers clues to the biology of PTSD, which sensitizes the brain to produce life-like flashbacks of a traumatic event.

## Case in Point: Schizophrenia

Of all the environmental contributions to mental illness, few are as mysterious as those in schizophrenia, which produces hallucinations, delusions, and paranoia, and accounts for nearly half the suicides among U.S. adolescents and young adults. Genetic factors drive much of the risk; those with schizophrenic relatives in their immediate family face a roughly tenfold greater likelihood of developing the disorder themselves.

But environmental threats also play a role. Some of the most persuasive data linking schizophrenia to environmental factors involve circumstances at birth. Urban birth, for instance, was shown to be linked to schizophrenia as far back as the 1930s by Robert E. Lee Faris and H. Warren Dunham, in classic studies that found high rates of the disease among children born in inner-city Chicago. Those same findings have since been replicated numerous times in several countries, such that researchers now routinely assume that urban birth raises the baseline risk of schizophrenia by roughly 50%. Other environmental risk factors vary widely, and include being born in winter and spring, maternal psychological stress during pregnancy, and obstetric complications.

What at least some of these factors might share in common, Brown suggests, are heightened exposures to infectious agents, which may be more common in inner cities or during colder months when the population is more likely to be sick. Studies have linked schizophrenia and prenatal exposure to a number of microbial infections, including those caused by rubella, toxoplasmosis, and influenza. Research led by Brown and Susser, described in the August 2004 issue of the *Archives of General Psychiatry*, shows that exposure to influenza *in utero* can raise the risk of schizophrenia. Brown has also demonstrated that inflammatory cytokines—such as interleukin 8, which is expressed at higher levels in the serum of mothers whose children later became schizophrenic—might somehow be involved. “We are presently examining how infection is related to cytokine disturbances in schizophrenia,” he says.

Susser, meanwhile, is also focusing his efforts on another compelling possibility—that schizophrenia can be triggered during pregnancy by maternal starvation. Research published in the 2 August 2006 issue of *JAMA* centered on two historical cohorts: a World War II–era famine in the western Netherlands caused by a Nazi blockade, and the Chinese famine that occurred from 1959 to 1961, precipitated by Mao Zedong’s disastrous Great Leap Forward policy. Evidence from both cohorts suggests that maternal famine can double the risk of schizophrenia among offspring.

“It *could be that these [gene–environment] interactions are more complex than our current knowledge allows us to imagine.”*

–Douglas Levinson, Stanford University School of Medicine

Together with Mary-Claire King, a professor in the departments of Genome Sciences and Medicine at the University of Washington, Susser is now investigating whether nutritional deficiencies might produce *de novo* mutations or epigenetic effects in genes required for normal brain development. One possibility is that folate deprivation such as occurs during famine might serve to inhibit DNA repair or alter DNA methylation. Susser and King have joined David St. Clair of Aberdeen University and Lin He of Jiaotong University to start a new study in China to explore this and other possible mechanisms.

Unlike *cancer or heart disease, which have clearly visible end points, mental disorders yield vague behaviors that vary widely among individuals.*

One potential environmental contributor has largely been ruled out as a cause of schizophrenia. Scientists used to think dysfunctional families were key risk factors, in part because they could expose children to “double-bind” interactions, such as a mother expressing love for her child verbally while she turns away in disgust. The emphasis on family dysfunction has since declined, however. More recent evidence, says Preben Bo Mortensen, a psychiatric epidemiologist at the Institute for Basic Psychiatric Research at Århus University Hospital, suggests that while dysfunctional families exacerbate the disease, they probably don’t trigger it.

## Prevention Opportunities

Susser suggests that recent findings regarding schizophrenia raise hope for its prevention, which has so far proven elusive. “That’s the big dream,” he says. “If we can show that supplementing with folate reduces risks, that would have real public health implications. The same applies if we can prevent the disease by limiting exposure to certain [toxicants] or infections. There’s lots of excitement about the possibilities, if we can specify how these pathways work.”

But environmental interventions aren’t beneficial just at the primary level. Scientists have also shown that secondary interventions, which remove environmental threats during early stages of mental illness, can sometimes reverse the course of a given disorder.

William McFarlane, a psychiatrist and researcher at Maine Medical Center, works with young people who show early warning signs for psychosis, including mild hallucinations and difficulty concentrating. Untreated, these individuals can progress to full-blown schizophrenia, characterized by extreme delusions and paranoia. But with a regimen of intense individual and family counseling and a range of supports at school and at work, combined with low doses of antipsychotic drugs, McFarlane’s patients learn how to identify and manage the stress triggers that heighten their mental instability. Over time, McFarlane says, they may outgrow their vulnerability to the disease, and go on to lead normal lives.

Unpublished preliminary data show that McFarlane’s approach can cut schizophrenia risk among vulnerable patients by half. In April 2007, he was awarded $12.4 million by the Robert Wood Johnson Foundation to expand his schizophrenia prevention program—ongoing in Portland, Maine, since 2000—to four additional cities nationwide.

Similarly, Shelley E. Taylor, a professor of psychology at the University of California, Los Angeles, has found that a positive, low-stress family environment lowers the risk of depression among children who harbor the variant serotonin transporter gene. More specifically, her research in the 1 October 2006 issue of *Biological Psychiatry* shows the variant’s effects in children could be amplified by cold, unsupportive family environments marked by conflict and anger, whereas warm, nurturing families were shown to counteract the variant gene.

Interestingly, environment-based interventions in mental illness could produce health benefits extending far beyond psychiatry. Studies consistently show that mental disorders elevate risks for a host of other health problems. Depression, for instance, increases health risks for heart failure patients, possibly by promoting the development of blood vessel plaques, according to research in the February 2007 *Archives of General Psychiatry* by Jesse Stewart, an assistant professor of psychology at Indiana University–Purdue University Indianapolis. Another recent study linked excessive anger and hostility in children—evident among those with antisocial personality disorders—to compromised lung function. That study, led by Benita Jackson of Smith College**,** appeared in the May 2007 issue of *Health Psychology*.

## Trends Unknown

With environmental research on mental health advancing, one key question remains unanswered: Are psychiatric disorders on the rise? Experts admit they don’t know. Kessler says cross-national trends are almost impossible to discern because of the varied ways mental health data are collected in different countries. “The data show almost no one in Nigeria is mentally ill, but no one in Nigeria talks about mental illness, so what are we to make of that information?” he asks. “Same in Japan—you find very low recorded rates of mental illness, but very high rates of suicide. So, clearly there’s something going on there that’s not making its way into the data.”

Trends in the United States have been hard to pick up because attitudes about mental illness have changed over time. Kessler is currently investigating differences in how Americans have responded to mood surveys from the 1950s onward. He anticipates a big increase in depression. The WHO concurs, predicting that by 2020, depression will be the second greatest contributor to the global burden of diseease (as measured by disability-adjusted life years) for all ages and both sexes. Kessler adds that any growth rate observed could merely reflect that people are more willing to talk about their feelings now than in times past, when such conditions carried a heavy stigma.

Torrey says prevalence data on mental illnesses are almost nonexistent in the United States. The most visible signs of a possible rise in mental health problems, he says, can be found among the homeless (of which nearly one-third are mentally ill) and in the nation’s jails and prisons (where up to half the inmates have psychiatric disorders), according to 2007 figures from the Federal Bureau of Justice Statistics.

The best prevalence data, Torrey says, come from Scandinavia, where researchers keep detailed registries of psychiatric admissions linked to national databases that track personal information relating to citizens. One of the most comprehensive systems of this kind exists in Denmark. But even there, the data don’t show any definitive trends, Mortensen says. “Looking at schizophrenia, for instance, we see what looks like a decrease until the early nineties, then an increase, and then a stabilization. But we think that most of those changes are related to diagnostic artifacts.” That is, the increase in diagnoses may reflect changes in diagnostic criteria, rather than changes in actual incidence.

In the end, one of the most positive developments to come from research into the biology of mental illness is a reduction in stigma. Throughout history, says Levinson, mentally ill patients have been shunted to the sidelines in medicine, the most severely among them institutionalized, while others struggle to survive in society. And because their ailments haven’t been linked to any obvious biological problems, patients have often been blamed for their conditions.

But now, that stigma appears to be on the decline, especially as physiological biomarkers for mental illness—for instance, changes in brain structure—begin to emerge. Ideally, growing recognition of mental illness as a biological phenomenon will fuel efforts to meet ongoing needs for adequate treatment, and as importantly, for environmental interventions that might serve to cost-effectively prevent large numbers of cases.

## obesity stress

A mouse study in the July 2007 issue of *Nature Medicine* shows that repeated stress in combination with a high-fat/high-sugar diet causes the release of the hormone neuropeptide Y (NPY), which in turn causes a buildup of abdominal fat. NPY is an appetite stimulant and growth factor that both enlarged fat cells and stimulated the production of new fat cells and blood vessels to support them. Each day, some of the mice stood in cold water for an hour, while others were exposed to an aggressive alpha male for 10 minutes. Stressed mice on a conventional diet had little variation in weight. But stressed mice on a high-fat/high-sugar diet accumulated twice as much abdominal fat in the first two weeks as unstressed mice on a conventional diet. Cold exposure not only increased circulating NPY but also, when combined with the poor diet, markedly upregulated expression of NPY in abdominal fat. The study is the first to show the importance of NPY’s role in obesity and metabolic syndrome.

Source: Kuo LE, Kitlinksa JB, Tilan JU, Li L, Baker SB, Johnson MD, et al. 2007. Neuropeptide Y acts directly in the periphery on fat tissue and mediates stress-induced obesity and metabolic syndrome. Nat Med 13(7):803–811.

## genes shyness

In the December 2005 issue of *Psychological Science*, researchers at the University of Maryland found that children with a particular variant of the serotonin transporter gene whose mothers reported low social support were more likely to be shy. However, if their mothers had plenty of social support, children with this variant were at no greater risk of shyness. The protein produced by the short form of the gene is known to predispose toward some forms of stress sensitivity (such as anxiety). In followup work published in the February 2007 issue of *Current Directions in Psychological Science*, the team found that mothers of naturally shy children may respond to their children in less nurturing fashion, reinforcing the children’s fearfulness and shyness.

Sources: Fox NA, Nichols KE, Henderson HA, Rubin K, Schmidt L, Hamer D, et al. 2005. Evidence for a gene–environment interaction in predicting behavioral inhibition in middle childhood. Psychol Sci 16(12):921–926.

Fox NA, Hane AA, Pine DS. 2007. Plasticity for affective neurocircuitry: how the environment affects gene expression. Curr Dir Psychol Sci 16(1):1–5.

## laughter allergies

Findings published in the June 2007 issue of the *Journal of Psychosomatic Research* suggest that when a mother laughs, her breast milk becomes enriched with melatonin that can ease allergic responses in her nursing infant. Laughter is known to increase natural killer cell activity in blood and free radicals in saliva, as well as reduce allergic skin wheal responses (the presence of raised, itchy patches) in patients with atopic eczema. The study included 48 infants with mild allergies to latex and dust mites. Half the mothers also had atopic eczema. When mothers watched a Charlie Chaplin film, breast milk concentrations of melatonin were significantly elevated up to eight hours after viewing, compared with the milk of mothers who watched a weather information film. Skin prick tests showed that infants who drank the melatonin-rich breast milk had a reduced wheal response.

Source: Kimata H. 2007. Laughter elevates the levels of breast-milk melatonin. J Psychosom Res 62(6):699–702.

## health costs parental depression

A study in the April 2007 issue of *Pediatrics* is the largest to date to show that children who have at least one depressed parent are more likely to use expensive health services. Researchers looked at patterns of health care use for nearly 70,000 Colorado children aged 3 months to 17 years. Nearly 25,000 of the children had at least one parent who had been diagnosed as having depression. Although teenagers of depressed parents had 5% fewer well-child visits, they were more likely to visit emergency departments and specialty clinics (including mental health, optometry, orthopedic, head/neck surgery, ophthalmology, dermatology, and allergy specialists). Infants with depressed parents had 14% more sick visits and 18% more emergency department visits than infants with nondepressed parents. More screening and treatment of parental depression would result in fewer emergency department visits and other expensive health care practices, and maternal screening during well child visits has already proven successful.

Source: Sills MR, Shetterly S, Xu S, Magid D, Kempe A. 2007. Association between parental depression and children’s health care use. Pediatrics 119(4):e829–e836.

## enzymes autism

Research in the April 2007 issue of the *Archives of Pediatric and Adolescent Medicine* showed a positive correlation between a diagnosis of autism in children and a polymorphism in a gene coding for the enzyme glutathione *S*-transferase in their mothers. These enzymes are involved in the detoxification of endogenous compounds such as peroxidized lipids, and in the metabolism of xenobiotic agents. The researchers determined the frequency of glutathione polymorphisms in 137 members of 49 families with a history of autistic spectrum disorders. Mothers of children with autism were 2.7 times more likely to carry the GSTP1*A haplotype. The results suggest that the haplotype “may be acting in mothers during pregnancy to contribute to the phenotype of autism in the fetus.”

Source: Williams TA, Mars AE, Buyske SG, Stenroos ES, Wang R, Factura-Santiago MF, et al. 2007. Risk of autistic disorder in affected offspring of mothers with a glutathione S-transferase P1 haplotype. Arch Pediatr Adolesc Med 161(4):356–361.

## alcoholism brain size

Alcoholics tend to have smaller brains than nonalcoholics, perhaps because ethanol causes the brain to shrink excessively with aging. A study published online in *Biological Psychiatry* on 15 February 2007 shows that brain size in alcoholics is also affected by their parents’ drinking, even before the alcoholic’s own dependence begins. Researchers used magnetic resonance imaging to measure intracranial volume (ICV)—a gauge for the lifetime maximum volume of the brain—in a group of people being treated for alcoholism. The average ICV of adult alcoholic children of alcoholic parents was about 4% smaller than that of adult alcoholics without family histories of alcoholism. Adult alcoholic children of alcoholic parents also had IQs averaging nearly 6 points lower than IQs of alcoholics with no parental drinking. The ICV of women in the study appeared to be affected more by their mothers’ drinking than their fathers’; this effect was not seen in the men in the study. The authors suggest that the increased risk for alcoholism among children of alcoholics may be due to a genetic or environmental effect, or both, related to reduced brain growth.

Source: Gilman JM, Bjork JM, Hommer DW. Parental alcohol use and brain volumes in early-and late-onset alcoholics. Biol Psychiatry 2007 Feb 15 [published online ahead of print].

## Figures and Tables

**Figure:**
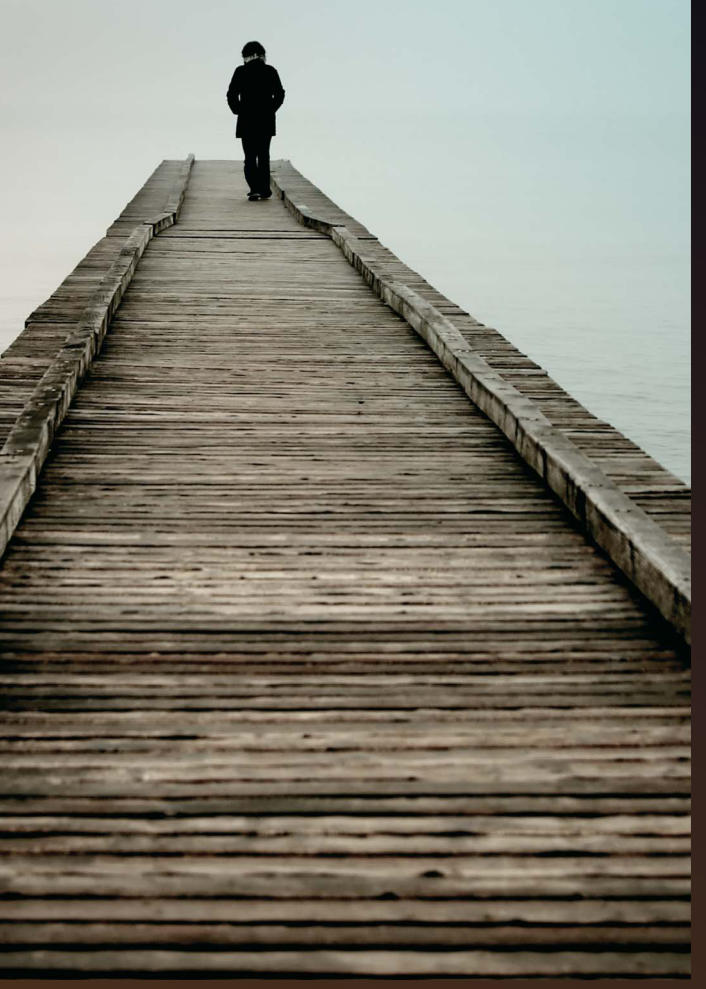


**Figure:**
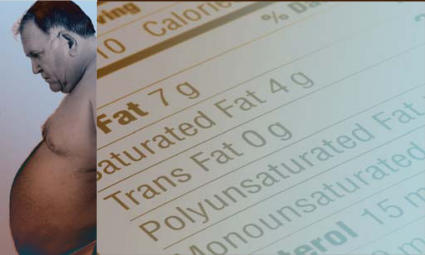


**Figure:**
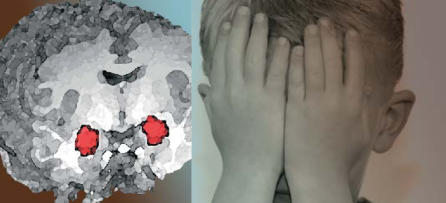


**Figure:**
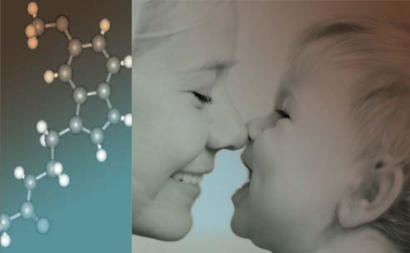


**Figure:**
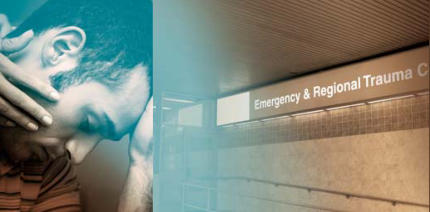


**Figure:**
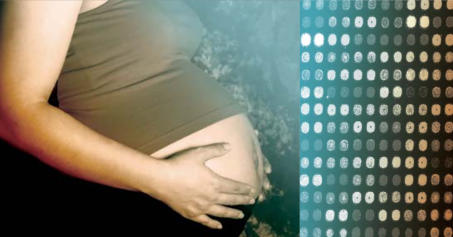


**Figure:**